# Effects of ALT-801, a GLP-1 and glucagon receptor dual agonist, in a translational mouse model of non-alcoholic steatohepatitis

**DOI:** 10.1038/s41598-022-10577-2

**Published:** 2022-04-23

**Authors:** John J. Nestor, David Parkes, Michael Feigh, John J. Suschak, M. Scott Harris

**Affiliations:** 1 Altimmune Inc, 910 Clopper Road, Suite 201S, Gaithersburg, MD 20878 USA; 2 DGP Scientific Inc., 156 Melanie Way, Del Mar, CA 92014 USA; 3 Gubra Aps, Hørsholm Kongevej 11B, 2970 Hørsholm, Denmark

**Keywords:** Non-alcoholic fatty liver disease, Non-alcoholic steatohepatitis

## Abstract

Body weight loss of ≥ 10% improves the metabolic derangements and liver disease in the majority of non-alcoholic steatohepatitis (NASH) patients, suggesting metabolic modulators may be effective in controlling disease. The pharmacodynamics of ALT-801, a GLP-1/glucagon receptor dual agonist optimized for NASH and weight loss, were compared to semaglutide (GLP-1 receptor agonist) and elafibranor (peroxisome proliferator-activated receptor, PPAR-α/δ, agonist) in a biopsy-confirmed, diet-induced obese (DIO) mouse model of NASH (DIO-NASH). Male C57BL/6J mice were fed Amylin Liver NASH (AMLN) diet for 32 weeks. Animals with biopsy-confirmed steatosis and fibrosis received ALT-801, semaglutide, elafibranor, or vehicle daily for 12 weeks while maintained on the AMLN diet. Study endpoints included body and liver weight, liver and plasma total cholesterol and triglycerides, plasma aminotransferases, histological analysis of liver steatosis, inflammation (galectin-3) and fibrosis (collagen type 1 alpha 1), and evaluation of individual animal changes in composite Non-alcoholic Fatty Liver Disease Activity Score (NAS), and fibrosis stage. ALT-801 demonstrated significant reductions in body weight (approx. 25%), plasma aminotransferases, plasma total cholesterol and liver triglycerides/total cholesterol in conjunction with improved liver steatosis, with greater reductions (*p* < 0.05) compared to semaglutide and elafibranor. ALT-801 significantly reduced the inflammation marker galectin-3 and the fibrosis marker collagen type 1 alpha 1 vs. vehicle (*p* < 0.05), with ALT-801 producing greater reductions in galectin-3 vs. elafibranor (*p* < 0.05). Importantly, all animals treated with ALT-801 significantly improved composite NAS compared to the active controls. This study provides evidence for a potential role for ALT-801 in the therapeutic treatment of NASH.

## Introduction

Non-alcoholic fatty liver disease (NAFLD) and non-alcoholic steatohepatitis (NASH) have long been considered liver manifestations of the metabolic syndrome, and their connection to obesity is well-recognized^1,2^. The expanding worldwide obesity epidemic^3^ and its comorbidities^4^ (cardiovascular disease, type 2 diabetes mellitus, extra-hepatic malignancies, NAFLD/NASH) have increased the importance of finding effective treatments for this obesity-associated spectrum of disease. A large and increasing range of therapeutic approaches are being assessed for the treatment of NAFLD/NASH, but most are directly targeting specific liver pathologies, while producing minimal to modest changes in body weight or composition^5^. While the use of glucagon-like peptide-1 (GLP-1) analogs in the control of diabetes has been associated with appetite suppression and reduced food intake, substantial numbers of patients exhibit gastrointestinal side effects (nausea, emesis) and average weight loss has ranged from 1 to 7% in studies leading to approval^6^. More recently the clinical use of GLP-1R agonist based therapeutics at high doses for the treatment of obesity and diabetes has resulted in weight loss in the 9–16% range over 26–68 weeks depending on diabetes status and the degree of lifestyle intervention^7,8^. For treatment of NASH, the reversal of fibrosis is a key unmet need and data suggests > 10% body weight loss is necessary for an optimal result^9^, an objective difficult to reach and maintain by lifestyle modification alone. Bariatric surgery remains the most effective route to achieve longer-term, definitive weight loss and reversal of obesity-driven comorbidities such as NAFLD/NASH^10,11^.

Beyond the GLP-1 receptor (GLP-1R) agonists, unimolecular peptide dual agonists activating both GLP-1R and the glucagon receptor (GCGR) have been developed with the aim to achieve superior therapeutic benefits versus single agonist peptides^12,13^. GLP-1R activation results in feeding suppression, while GCGR activation stimulates increased energy expenditure, adipose tissue browning, mobilization and metabolism of fat, leading to substantial body weight loss and clearance of liver fat in animal models^12–17^. Activation of GCGR also has direct effects on liver lipid metabolism and may act synergistically with GLP-1 in the treatment of obesity and NASH^18^. While one of the physiological roles of glucagon is to increase blood glucose in starvation states, posing a hyperglycemic risk, the simultaneous blood glucose-dependent potentiation of insulin secretion by GLP-1 can counteract this effect. In unimolecular GLP-1R/GCGR dual agonist studies^14–17^, the ratios of the GLP-1R and GCGR potencies have varied and typically have been significantly greater than 1:1 despite evidence that an evenly balanced, 1:1 ratio results in optimal metabolic effects^18^.

We recently reported studies examining a novel glycolipid surfactant-peptide conjugation technology designed to prolong in vivo terminal elimination half-life (T_1/2_) and demonstrated its tunable impact on pharmacokinetic (PK) parameters for analogs of both parathyroid hormone^19^ and GLP-1R/GCGR dual agonists^20^. We identified ALT-801 (previously known as SP-1373, Spitfire Pharma, Inc.), as a potent, long-acting, and evenly balanced agonist of the GLP-1R and GCGR that was optimized for NASH and weight loss^20,21^. In studies of ALT-801, the prolonged duration of action in vivo, comparable to the literature standard semaglutide (GLP-1R agonist), suggests the suitability of ALT-801 for once weekly administration to patients. Here we report on an in-depth pharmacology study with ALT-801, a dual GLP-1R/GCGR peptide agonist which is conjugated to a novel glycolipid moiety to slow absorption of the peptide into the plasma and prolong its half-life. We evaluated ALT-801 in a translational, biopsy-confirmed diet-induced obese (DIO) mouse model of NASH (DIO-NASH)^22^ and investigated the effects of a 12 week treatment regimen on metabolic and biochemical parameters, hepatic pathology, and individual changes in NAFLD Activity Score (NAS) and fibrosis stage.

## Results

### ALT-801 pharmacokinetics

**Figure 1:**
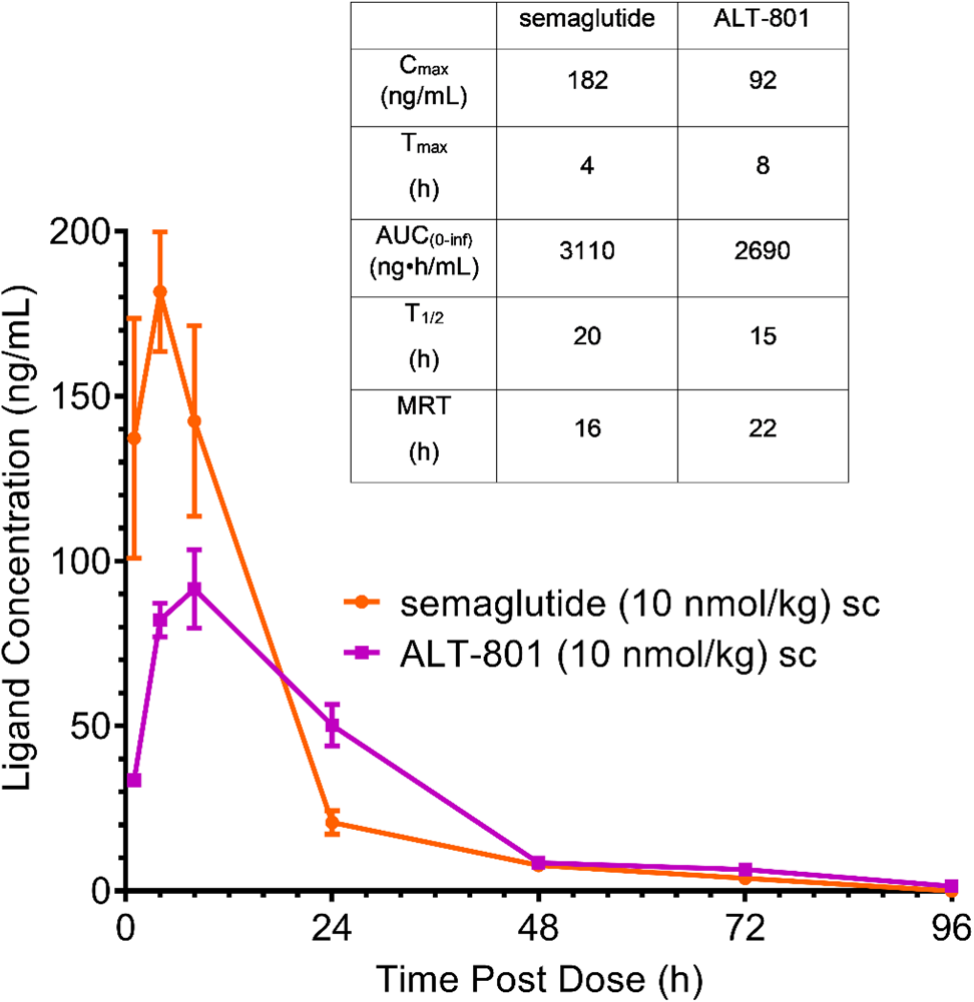
Pharmacokinetic behavior of ALT-801 and semaglutide in C57Bl/6J mice following subcutaneous administration. Mice received a single s.c. injection of ALT-801 or semaglutide at 10 nmol/kg and key PK characteristics were calculated. Compared to semaglutide, for ALT-801 the data indicate a longer T_max_ with a substantially lower C_max_ and an overall delayed PK envelope, having a similar AUC.

PK parameters for equimolar doses of ALT-801 (10 nmol/kg; 39 µg/kg) and semaglutide (10 nmol/kg; 41.1 µg/kg) administered via the s.c. route are indicated in Fig. 1 (T_max_ = 8 and 4 h, C_max_ = 92 and 182 ng/mL, MRT = 22 and 16 h; respectively) and suggest a more measured and delayed approach to C_max_ in mice treated with ALT-801 relative to semaglutide. ALT-801 had a C_max_ 50% of, but AUC > 86% of, the values for semaglutide, the literature standard. Elafibranor PK parameters were not assessed as it required the oral route of administration and was therefore not comparable to ALT-801 or semaglutide given by the s.c. route.

### Treatment with ALT-801 decreases body weight in DIO-NASH mouse model

**Figure 2:**
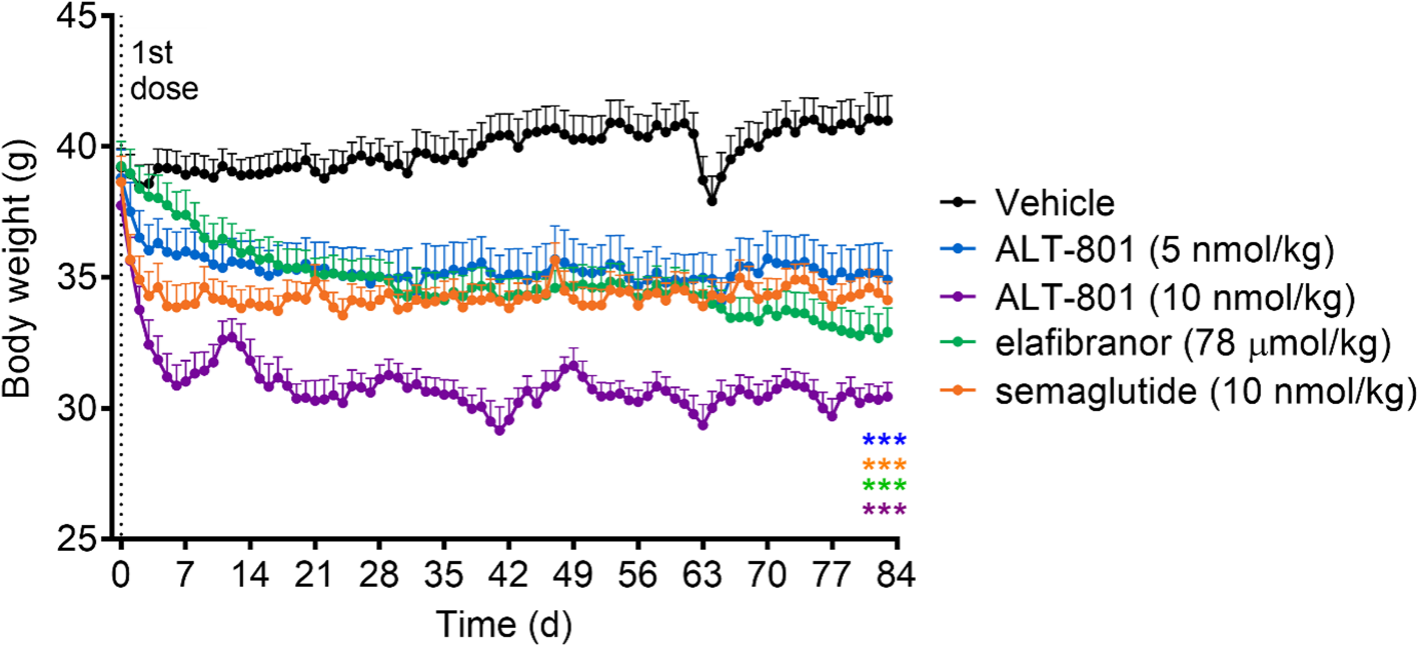
Body weight of treatment groups. DIO-NASH mice were treated daily with vehicle (s.c.), ALT-801 (5 and 10 nmol/kg, s.c.), elafibranor (78 μmol/kg, oral) or semaglutide (10 nmol/kg, s.c.). Body weight was recorded daily. For ALT-801 and semaglutide, rapid body weight decreases occurred within 1–2 weeks and stabilized. Elafibranor (PPAR-α/δ agonist) causes weight loss in rodents but not in clinical trials. On day 63 vehicle animals were mis-dosed with ALT-801 10 nmol/kg, causing a rapid ~ 8% body weight decrease, which resolved over a period of days. ALT-801 treatment (10 nmol/kg) reduced body weight to the lean normal range (~ 30 g; − 25%). Data are expressed as mean ± SEM (n = 11–12). ***p ≤ 0.001 vs. vehicle control.

In the DIO-NASH mouse model, treatment with ALT-801, semaglutide, or elafibranor caused significant (*p* < 0.001) body weight decreases that stabilized after 2–3 weeks and through the remainder of the study (Fig. 2). The weight loss achieved in animals treated with ALT-801 10 nmol/kg reached − 25% of vehicle control weights within 3 weeks of administration and was approximately twice the weight loss induced by semaglutide at the equimolar dose. Importantly, ALT-801 10 nmol/kg decreased the body weight for the group to the normal body weight range for this mouse strain (~ 30 g), then maintained this range^23,24^. Weight loss following treatment with elafibranor is known to occur in rodent models but is generally not observed clinically^25,26^. During treatment week 9 (day 63) the vehicle group was inadvertently given a single dose of ALT-801 10 nmol/kg, resulting in a rapid decline in weight, illustrating the high potency and efficacy of ALT-801. The control animals recovered the lost body weight over a period of days and returned to prior weight levels by study termination.

### Treatment with ALT-801 improves hepatomegaly and hepatic steatosis in a DIO-NASH mouse model

**Figure 3:**
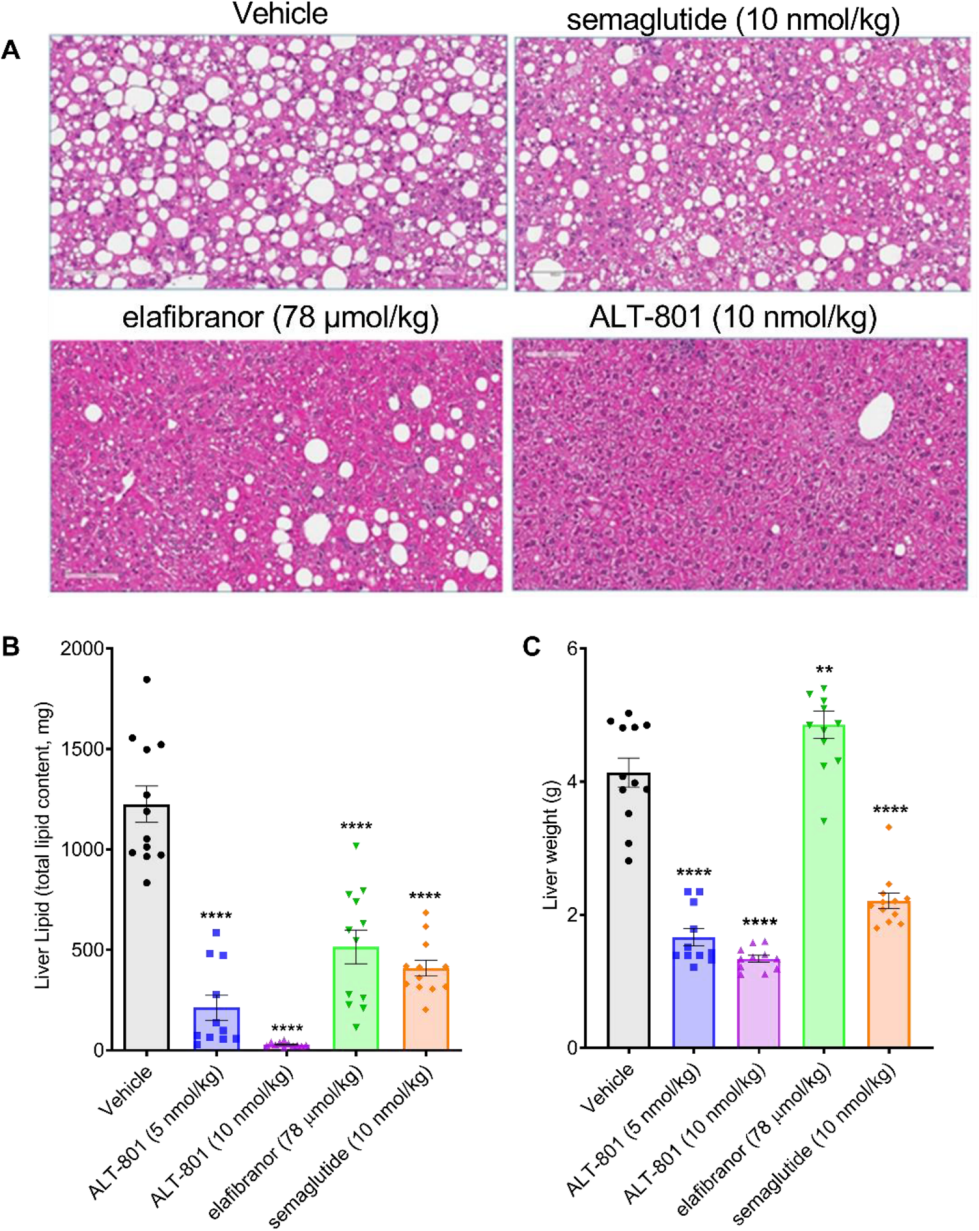
Effects of treatment on liver fat content and liver weight. (**A**) Representative H&E-stained images of liver morphology at the end of treatment period for vehicle, semaglutide, elafibranor, and ALT-801 10 nmol/kg treated DIO-NASH animals (magnification 20×, scale bar = 100 µm). (**B**) Mean total liver fat content (mg). (**C**) Mean terminal liver weight (g). Data are expressed as mean ± SEM (n = 11–12). **p < 0.01, ****p < 0.0001 vs. vehicle control.

At the end of the dosing period, each of the treatments resulted in a significant (*p* < 0.0001) reduction in liver fat content relative to vehicle (Fig. 3A). Liver fat reduction following treatment with ALT-801 10 nmol/kg was significantly greater than with 5 nmol/kg ALT-801, semaglutide, or elafibranor (*p* < 0.005), resulting in a near normal appearance histologically for the high dose group (Fig. 3B). Low and high dose treatment with ALT-801 also resulted in significantly decreased liver weight as compared to vehicle control (*p* < 0.0001), and to semaglutide and elafibranor (*p* < 0.001) (Fig. 3C), resulting in a near normal liver weight for the high dose group.

### Treatment with ALT-801 improves NAS and fibrosis stage in a DIO-NASH mouse model

**Figure 4:**
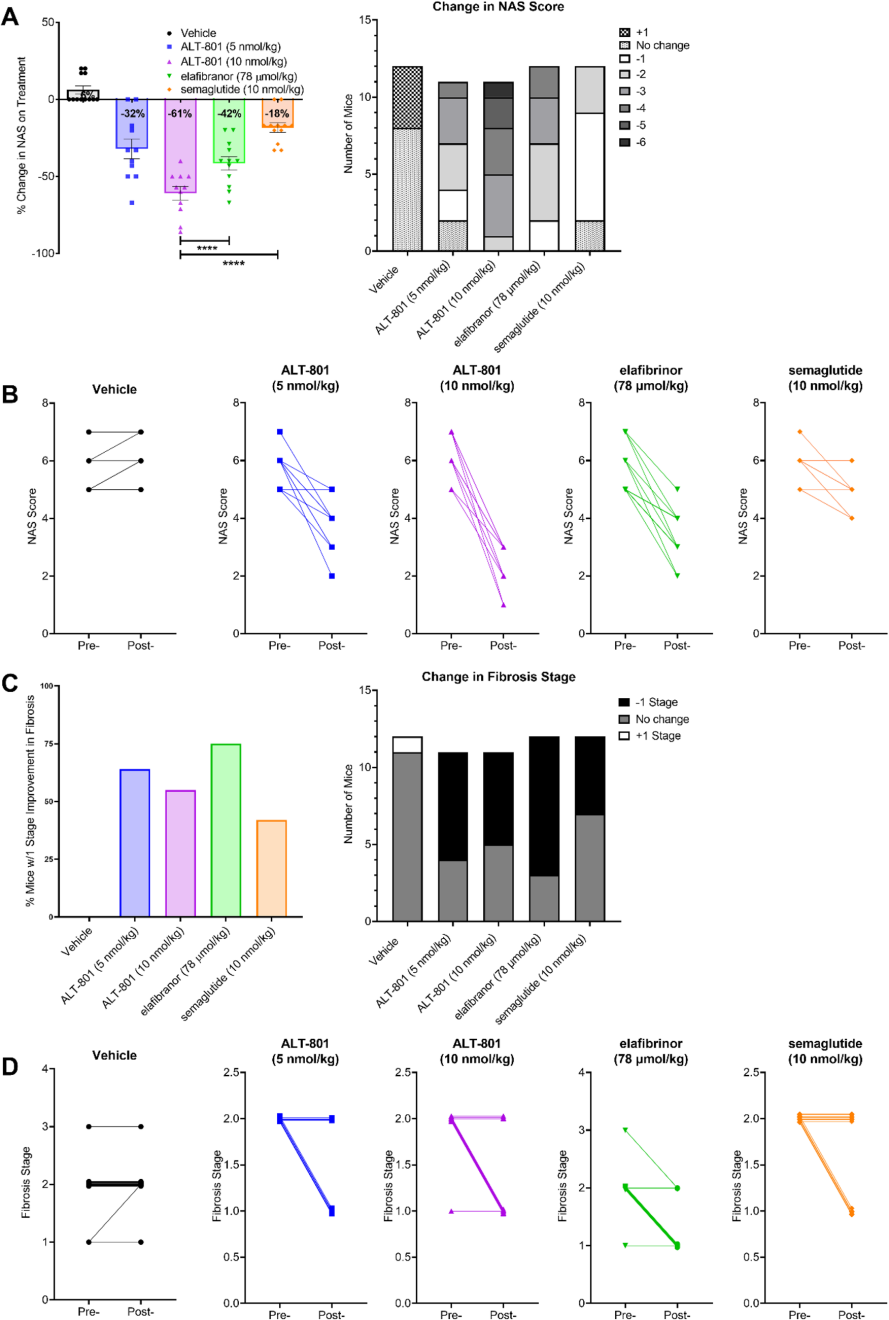
Treatment effects on NAFLD Activity Score and fibrosis stage. (**A**) Change in mean non-alcoholic fatty liver disease activity score (NAS). Significance is vs. ALT-801 10 nmol/kg. (**B**) Change in individual NAS. (**C**) Change in mean fibrosis stage. (**D**) Change in individual fibrosis stages. The points at each scoring step are slightly shifted to allow visual separation of the animals. This presentation is only for visualization purposes and does not reflect any difference in score. ****p < 0.0001.

The NAS improved in all treatment groups at the end of the treatment period compared to vehicle (Fig. 4A,B), with a change in NAS of − 32% and − 61% in the low and high ALT-801 treatment groups, respectively, compared to the start of treatment (day 0). Elafibranor and semaglutide treatment groups experienced a − 42% and − 18% change, respectively, compared to the start of treatment, and the vehicle control group experienced a 6% increase. The percent change in NAS achieved by the elafibranor and semaglutide treatment groups were significantly less than the percent change achieved in the ALT-801 10 nmol/kg group (both *p* < 0.0001). All animals in the ALT-801 10 nmol/kg group achieved a reduction in NAS of at least 3. Changes in the component elements of steatosis, inflammation and hepatocyte ballooning for each of the study groups can be found in Supplementary Figs. S1–S4.

Fibrosis stage also improved in all treatment groups at the end of the treatment period compared to vehicle (Fig. 4C,D). Compared to the start of the treatment, reduction in fibrosis stage of 1 or greater was observed in 64%, 55%, 75% and 42% of the animals in the low ALT-801, high ALT-801, elafibranor and semaglutide groups respectively, while none of the animals in the vehicle group showed any improvements.

### Treatment with ALT-801 improves hepatic markers for inflammation and fibrosis and lipid content in a DIO-NASH mouse model

**Figure 5:**
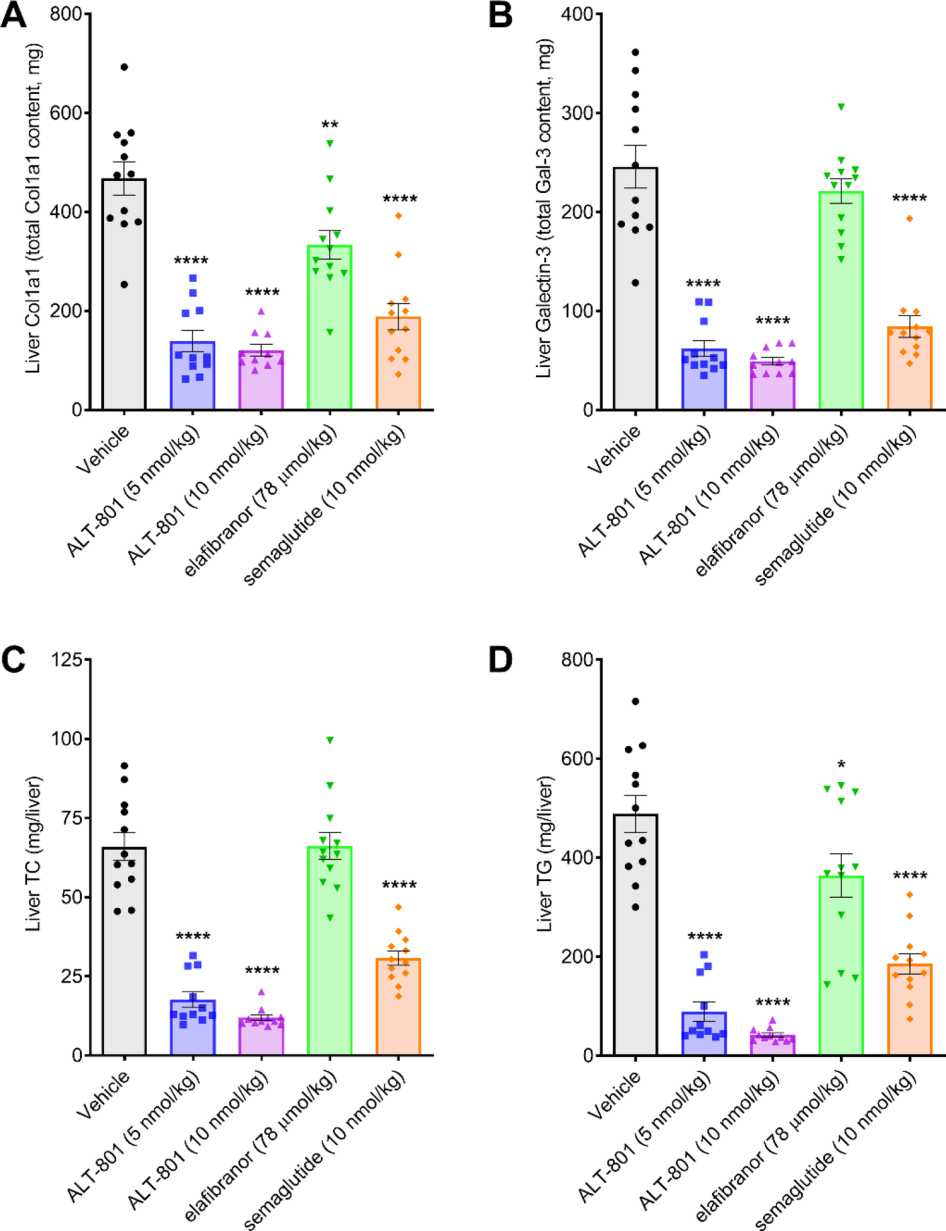
Treatment effects on liver inflammation, fibrosis and lipids. Shown are changes in liver histological and biochemical markers following 12 weeks of treatment with the study drugs. (**A**) Change in terminal liver Col1A1. (**B**) Change in terminal liver Gal-3. Quantification of Col1A1 and Gal-3 was determined by histomorphometry. (**C**) Total liver cholesterol (TC) content. (**D**) Total liver triglycerides (TG). Data are expressed as mean ± SEM (n = 11–12). *p < 0.05, **p < 0.01, ****p < 0.0001 vs. vehicle control.

Low and high dose treatment with ALT-801 resulted in a significant reduction in the inflammation marker Gal-3 and the fibrosis marker Col1A1 (Fig. 5). All data are presented on a whole liver basis in view of the very different terminal liver weights for different compounds. Both doses of ALT-801 led to significantly greater decreases in liver galectin-3 content compared to elafibranor (*p* < 0.05).

Low and high dose treatment with ALT-801 also resulted in significantly lower total liver TC (*p* < 0.0001) and TG (*p* < 0.0001) compared to vehicle control. Both doses of ALT-801 also resulted in significantly lower total liver cholesterol levels compared to semaglutide and elafibranor (*p* < 0.0005).

### Treatment with ALT-801 reduces circulating liver enzymes and total cholesterol in a DIO-NASH mouse model

**Figure 6:**
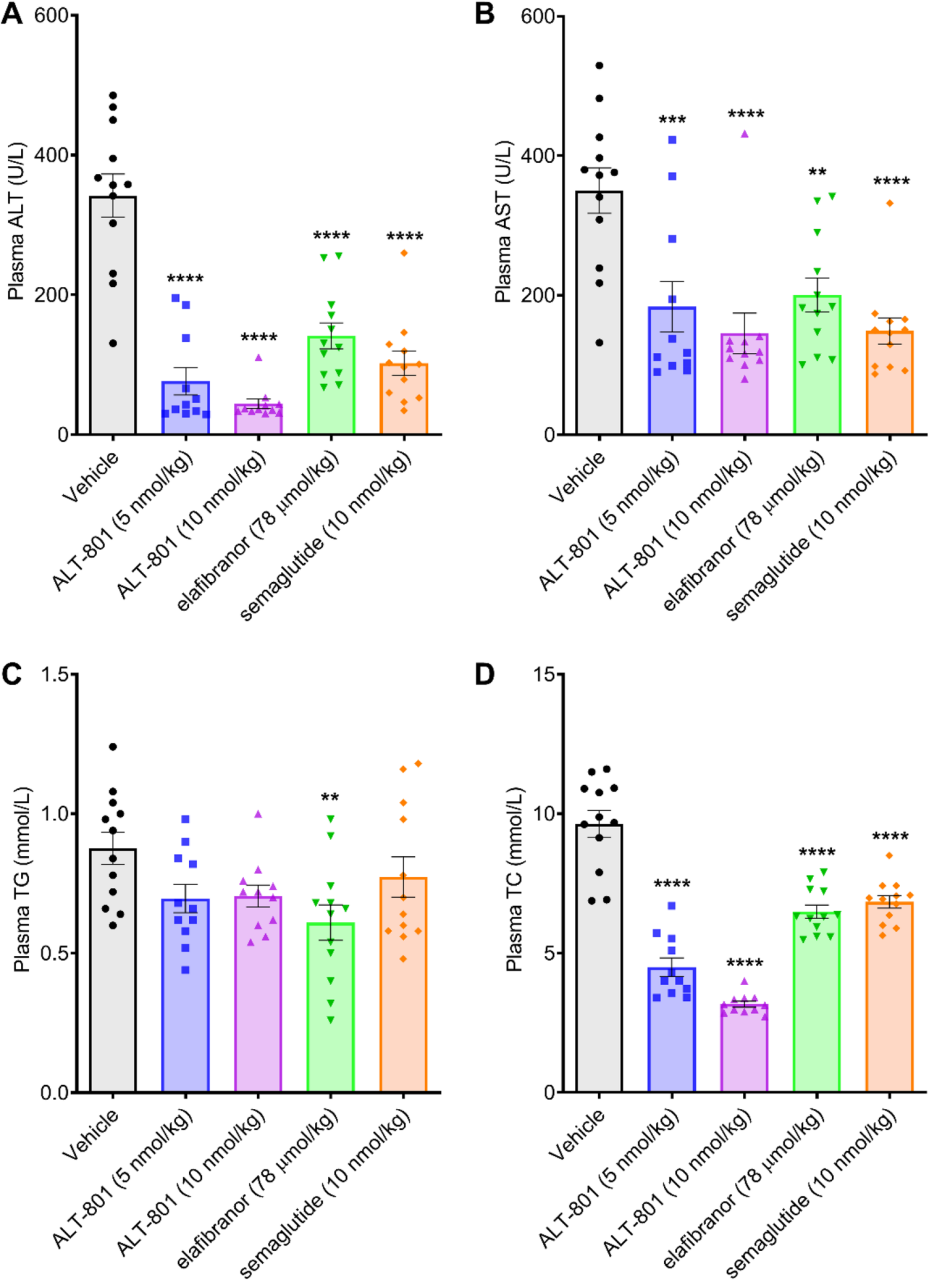
Effects of treatment on plasma liver markers. (**A**) Plasma ALT. (**B**) Plasma AST. (**C**) Plasma TG. (**D**) Plasma TC. Data are expressed as mean ± SEM (n = 11–12). One-way ANOVA with Dunnett’s adjustment for multiplicity. **p < 0.01, ***p < 0.001, ****p < 0.0001 vs. vehicle control.

Low and high dose treatment with ALT-801 also had profound effects on circulating liver enzymes, including terminal plasma ALT and AST levels, which were significantly lower compared to vehicle control (*p* < 0.001), and significantly lower than that achieved with elafibranor (*p* < 0.05; Fig. 6). Importantly, plasma ALT levels were returned to the normal range with ALT-801 (10 nmol/kg). Plasma TC was similarly reduced in all treatment groups, but the reduction was significantly greater in both of the ALT-801 groups than in either the semaglutide or elafibranor groups (*p* < 0.0001). Not surprisingly, given the lipid-mobilizing, catabolic effects of glucagon, plasma TG levels were lower in the ALT-801 groups compared to the vehicle control and semaglutide groups, but the differences did not reach statistical significance.

## Discussion

Following 12 weeks of treatment in a biopsy-confirmed, diet-induced obese mouse model of NASH, ALT-801, a GLP-1R/GCGR dual agonist, was shown to significantly lower body weight, plasma cholesterol and liver enzymes, liver weight, liver steatosis, histological markers of inflammation and fibrosis and, importantly, composite NAS, compared to vehicle-treated control. Remarkably, most of these parameters improved to the normal lean (not shown) range while the fibrosis improvement was comparable to semaglutide and elafibranor treatments. In addition, the study revealed that PK properties of ALT-801 were comparable to those of semaglutide and therefore consistent with a weekly dosing schedule and similar exposure for patients, but with a delayed T_max_ and lowered C_max_, which may provide reduced peak to trough ratios during treatment.

Treatment of DIO-NASH mice with ALT-801 resulted in greater reductions in most measures of NASH compared to either semaglutide or elafibranor, and ALT-801 administration significantly reduced the inflammatory marker Gal-3 and fibrosis marker Col1A1, with Gal-3 reduction at a greater degree than either comparator drug. All DIO-NASH animals treated with ALT-801 improved composite NAS to ≤ 3, which was driven by reductions in steatosis, lobular inflammation, and hepatocellular ballooning scores. The extent of clearance of hepatic steatosis with ALT-801 is particularly striking in comparison to the GLP-1R only agonist, semaglutide (Fig. 3).

Equivalent doses of ALT-801 and semaglutide were used throughout this study. While an approximately seven-fold higher dose of semaglutide (based on allometric scaling) was recently approved for weight loss in overweight and obese individuals, the 12% weight loss effected by semaglutide in this study is similar to that observed clinically with high dose semaglutide^7^ supporting the dose used in this study. Elafibranor has been employed here as a positive control at the dose used in other rodent models of NASH^42,43^.

While previous studies with investigational NASH agents that have reached clinical testing have reported body weight, liver weight, plasma lipids, AST, ALT, or histological findings^27–30^, only GLP-1 based approaches have been associated with significant weight loss in published trials to date. Significant improvement of NASH has been associated with greater than 5% weight loss, but resolution of fibrosis appears to require > 10% body weight loss for optimal effects^9,11,31–33^. In this context the pronounced weight loss effects observed with ALT-801, together with the demonstrated effects on the other NASH indices reported here, whether directly or indirectly resulting from weight loss, is especially important. It is worth noting that while GCGR are present in livers of rodents and primates, GLP-1R are not^34,35^.

The chemical structure of ALT-801 utilizes a stabilized peptide structure including sequence elements of both GLP-1 and glucagon, coupled with EuPort™ modification, a proprietary glycolipid moiety designed to achieve a prolonged duration of action consistent with weekly dosing^20^. The glycolipid surfactant, which provides near quantitative but transient binding to serum albumin (estimated as > 99%), helps prevent proteolysis and clearance by glomerular filtration. In addition, the surfactant-like features of the EuPort™ domain lead to formation of micelles, which, upon s.c. administration, slow the release of the drug substance into the blood stream, as evidenced by the reduced T_max_ and C_max_ and prolonged AUC. While glycolipid surfactant conjugation has not been used previously in peptide design, injection site depot formation and serum albumin binding are recognized design approaches and have similarities to earlier drug designs^36–39^.

Pharmacokinetic assessment of ALT-801 in mice demonstrated reduced C_max_ but with similar AUC_0–inf_ values (50% and > 86%, respectively) compared to the semaglutide parameter values. These parameters are potentially attractive in that there is potential for lowered peak to trough ratio during dosing^40^ and therefore potential for a decreased gastrointestinal side effect profile for ALT-801 compared to those typically seen with GLP-1 agonists^41^.

Results of this study indicate that a GLP-1R/GCGR dual agonist (ALT-801) performed better than GLP-1R and PPAR-α/δ agonists in treating most NASH parameters. Seminal studies in DIO mice demonstrated that GLP-1R/GCGR dual agonists had improved effects on weight loss compared to GLP-1R agonists, while maintaining glucoregulatory action^12,13,18^. Moreover, ALT-801 is a balanced dual receptor agonist^19,20^ with closely matched potencies at the GLP-1R and GCGR (39 pM and 42 pM, respectively^20^), as distinguished from the dual agonists cotadutide^42^ and BI456906^43^, which appear to be biased 5:1 and 7.5:1, respectively, toward GLP-1R activation. A study that evaluated GLP-1R/GCGR dual agonists of varying ratios of potency at the GLP-1R and GCGR, in essence evaluating the pharmacodynamic effects of dual agonists that were either balanced in their receptor activation potencies or biased toward one receptor or the other, indicated that a dual agonist with near balanced activity at GLP-1R and GCGR was associated with the greatest weight loss and with glycemic control^18^. The potency and efficacy of ALT-801 is further demonstrated by the remarkable weight loss effected by a single dose of 10 nmol/kg to the vehicle group on day 63 (Fig. 2). Given this background and the fact that the apparent potency of ALT-801 for the GLP-1R is comparable to that of semaglutide (EC_50_ of 39 pM and 15 pM, respectively^20^) it is reasonable to conclude that GCGR activation is responsible for the improved activity of ALT-801 over semaglutide in this NASH model.

Similarly, another study compared body weight, food consumption, and total energy expenditure outcomes for GLP-1R/GCGR dual agonist and a GLP-1R agonist in DIO mice and DIO monkeys^16^. Results of this study indicated that both agonists improved glycemic control; however, the dual agonist elicited a greater reduction in body weight in both species relative to the GLP-1R agonist alone. Notably, the dual agonist was able to elicit greater body weight loss in DIO monkeys at a tenfold lower dose than the GLP-1R agonist, demonstrating greater efficacy. That study also demonstrated that a GLP-1R/GCGR dual agonist induced body weight loss across species, which is promising for ALT-801 development to treat NASH and the accompanying liver fibrosis. ALT-801 (denoted as Compound 17) has also been shown to reduce blood glucose in the diabetic *db/db* mouse model at least as well as an equivalent dose of semaglutide^20^, suggesting that the glucoregulatory effects of the GLP-1R activation by ALT-801 were not disrupted by simultaneous agonism of the GCGR. While previous studies have compared GLP-1R/GCGR and GLP-1R agonists, the current study appears to be the first to directly compare a GLP-1R/GCGR and a PPAR-α/δ agonist, demonstrating statistically improved activity of ALT-801 over elafibranor in most NASH measures.

Overall, the potential for lower side effects related to lower C_max_ values, coupled with improvements of body weight, liver pathology, and metabolic parameters in this DIO-NASH mouse model, highlight ALT-801 as an attractive new drug candidate for the treatment of NASH and human clinical studies are underway.

## Methods

### Pharmacokinetics

PK parameters following a single subcutaneous (s.c.) administration of ALT-801 or semaglutide (both stock concentrations at 10 nmol/kg) were evaluated in male C57BL6/J mice at The Jackson Laboratory-JAX West (Sacramento, CA). Both compounds were formulated at 0.02 mg/mL in 50 mM phosphate buffer and 0.05% Tween 80 at pH ~ 8. The dosing volume was approximately 2 mL/kg. Blood samples (~ 200 µL) were collected at 1, 4, 8, 24, 48, 72, 96, and 120 h post-dosing (n = 4 per time point). Each mouse was bled at two time points and the second time point was a terminal bleed. Plasma concentrations of ALT-801 and semaglutide were determined using liquid chromatography coupled with tandem mass spectrometry with a limit of quantitation of 1 and 2 ng/mL for semaglutide and ALT-801, respectively. Non-compartmental PK analysis using WinNonlin was performed by using the mean concentrations at each sampling time point to report the C_max_, T_max_, the area under the plasma concentration curve from time zero to infinity (AUC_0–inf_), T_1/2_, and the mean residence time (MRT).

### Biopsy-confirmed DIO-NASH mouse model

ALT-801 was evaluated for its metabolic and anti-fibrotic effects during 12 weeks of treatment in the well-documented Amylin Liver NASH (AMLN) DIO-NASH mouse model^22,44^ at Gubra ApS. Mice had ad libitum access to tap water and a diet high in fat (40%, containing 18% trans-fat; 40% carbohydrates, 20% fructose) and 2% cholesterol (AMLN diet; D09100301, Research Diets, New Brunswick, NJ). Semaglutide (GLP-1R agonist) and elafibranor (peroxisome proliferator-activated receptor [PPAR]-α/δ agonist) were included as comparators to assess the effects of added GCGR agonism, and a previously observed modest antifibrotic effect in this assay, respectively^45^.

All experiments were conducted in accordance with Gubra Aps’ bioethical guidelines, which were fully compliant with internationally accepted principles for the care and use of laboratory animals. The Danish Animal Experiments Inspectorate approved all experiments which were conducted using internationally accepted principles for the use of laboratory animals under the personal license #2013-15-2934-00784. We also confirm that the study was carried out in compliance with the ARRIVE guidelines. Sixty male, 5-wk old, wild-type C57BL/6J mice (JanVier Labs, France) were group housed in a controlled environment (12 h light–dark cycle, 21 ± 2 °C, 50 ± 10% humidity) and fed the AMLN diet high in trans-fat, cholesterol, and fructose^46^ ad libitum for 32 weeks to induce obesity and the NASH phenotype.

After 29 weeks of diet induction (week-3 relative to start of treatment) a pre-treatment liver biopsy was performed as described in detail previously^44^ to select animals for stratification and randomization based on histological assessment. Liver histology was scored using the NAS and fibrosis stage criteria defined by the NASH Clinical Research Network Pathology Committee^47^ and only those animals with confirmed steatosis (score ≥ 2) and fibrosis (stage ≥ 1) were selected for stratification and randomization. A stratified randomization into treatment groups was performed for the biopsy-proven DIO-NASH mice according to liver collagen type 1 alpha 1 (Col1A1) quantification at week-3. Test animals (n = 12/group) were then single housed and treated once daily for a period of 12 weeks while maintaining the AMLN diet. Treatment groups were vehicle (0.05% Tween 80 in 50 mM Na_2_HPO_4_, pH 8, s.c.), 10 nmol/kg semaglutide (0.02 mg/mL, s.c.), 5 nmol/kg ALT-801 (0.02 mg/mL, s.c.), 10 nmol/kg ALT-801 (0.02 mg/mL, s.c.), or 78 μmol/kg elafibranor (6 mg/mL in 0.5% carboxymethyl cellulose, oral). ALT-801 and semaglutide were synthesized (≥ 95% purity) and supplied as acetate salts by CS Bio Co. (Menlo Park, CA). Elafibranor was obtained from Sunshine Chemical (Wuhan, China). After a total of 12 weeks on treatment, the animals were euthanized, and liver tissue and terminal plasma were collected for histological and biochemical analysis.

### NAFLD Activity Score, fibrosis stage and histological assessment of steatosis, inflammation, and fibrosis

Paraffin embedded slides of biopsied baseline liver tissue (50 to 100 mg) and terminal samples (approximately 200 mg) were prepared to assess hepatic morphology including steatosis and fibrosis by staining with hematoxylin and eosin (H&E) and picrosirius red, respectively. Immunohistochemical staining for Col1A1 (baseline biopsy and terminal liver) and galectin-3 (Gal-3) (terminal samples only) was also performed to assess hepatic fibrosis and inflammation, respectively. Quantitative assessment of immunoreactivity was performed via image analysis using Visiomorph software (Visiopharm, Denmark). Blinded liver histology was scored using NAS criteria^47^ and fibrosis stage.

### Plasma biochemistry analysis

Terminal blood samples were collected in heparinized tubes and plasma was separated and stored at − 80 °C until analysis. Triglycerides (TG), total cholesterol (TC), alanine aminotransferase (ALT), and aspartate aminotransferase (AST) plasma levels were measured using commercial kits (Roche Diagnostics, Germany) on a Cobas C-501 auto analyzer according to manufacturer’s instructions.

### Terminal hepatic triglyceride and total cholesterol content

The TG and TC content in terminal liver samples (25 mg) was determined using the Triglyceride Reagent (Roche Diagnostics, Germany) and the Cholesterol reagent (Roche Diagnostics, Germany), respectively, on a Cobas C-501 auto analyzer. Homogenized liver tissue was heated to between 80 and 100 °C twice, centrifuged in a microcentrifuge, and the TG and TC content were measured in the supernatant.

### Statistical analysis

A one-way ANOVA with Dunnett’s multiple comparison test were used to assess statistical significance between groups. When comparing results between treatment groups, a one-way ANOVA with Tukey’s multiple comparison test was used. Statistical significance was designated at the conventional α level of *p* < 0.05.

## Supplementary Information

Supplementary Figures.

## Data Availability

The data that support the findings of this study are available from the authors on reasonable request pending approval from all relevant institutions.

## References

[1] Softic S, Kahn CR (2019). Fatty liver disease: Is it nonalcoholic fatty liver disease or obesity-associated fatty liver disease?. Eur. J. Gastroenterol. Hepatol..

[2] Eslam M, Sanyal AJ, George J, International Consensus, P. (2020). MAFLD: A consensus-driven proposed nomenclature for metabolic associated fatty liver disease. Gastroenterology.

[3] Afshin A, Reitsma MB, Murray CJL (2017). Health effects of overweight and obesity in 195 countries. N. Engl. J. Med..

[4] Glass LM, Hunt CM, Fuchs M, Su GL (2019). Comorbidities and nonalcoholic fatty liver disease: The chicken, the egg, or both?. Fed. Pract..

[5] Sumida Y, Yoneda M (2018). Current and future pharmacological therapies for NAFLD/NASH. J. Gastroenterol..

[6] Nauck MA, Meier JJ (2019). Management of endocrine disease: Are all GLP-1 agonists equal in the treatment of type 2 diabetes?. Eur. J. Endocrinol..

[7] Wilding JPH (2021). Once-weekly semaglutide in adults with overweight or obesity. N. Engl. J. Med..

[8] Frias JP (2021). Tirzepatide versus semaglutide once weekly in patients with type 2 diabetes. N. Engl. J. Med..

[9] Vilar-Gomez E (2015). Weight loss through lifestyle modification significantly reduces features of nonalcoholic Steatohepatitis. Gastroenterology.

[10] Ampuero J, Sanchez-Torrijos Y, Aguilera V, Bellido F, Romero-Gomez M (2018). New therapeutic perspectives in non-alcoholic steatohepatitis. Gastroenterol. Hepatol..

[11] Lassailly G (2020). Bariatric surgery provides long-term resolution of nonalcoholic steatohepatitis and regression of fibrosis. Gastroenterology.

[12] Pocai A (2009). Glucagon-like peptide 1/glucagon receptor dual agonism reverses obesity in mice. Diabetes.

[13] Day JW (2009). A new glucagon and GLP-1 co-agonist eliminates obesity in rodents. Nat. Chem. Biol..

[14] Patel V (2018). Coagonist of GLP-1 and glucagon receptor ameliorates development of non-alcoholic fatty liver disease. Cardiovasc. Hematol. Agents Med. Chem..

[15] Zhou J (2017). A novel glucagon-like peptide-1/glucagon receptor dual agonist exhibits weight-lowering and diabetes-protective effects. Eur. J. Med. Chem..

[16] Elvert R (2018). Running on mixed fuel-dual agonistic approach of GLP-1 and GCG receptors leads to beneficial impact on body weight and blood glucose control: A comparative study between mice and non-human primates. Diabetes Obes. Metab..

[17] Tillner J (2019). A novel dual glucagon-like peptide and glucagon receptor agonist SAR425899: Results of randomized, placebo-controlled first-in-human and first-in-patient trials. Diabetes Obes. Metab..

[18] Day JW (2012). Optimization of co-agonism at GLP-1 and glucagon receptors to safely maximize weight reduction in DIO-rodents. Biopolymers.

[19] Nestor JJ, Wang W (2021). Surfactant-modified parathyroid hormone fragments with high potency and prolonged action: Structure-informed design using glycolipid surfactant conjugation. Pept. Sci..

[20] Nestor JJ, Zhang X, Jaw-Tsai S, Parkes DG, Becker CK (2021). Design and characterization of a surfactant-conjugated, long-acting, balanced GLP-1/glucagon receptor dual agonist. Pept. Sci..

[21] Nestor, J. J., Jr., Zhang, X., Perlman, A., Parkes, D. G. & Jaw-Tsai, S. In *Long-Acting GLP-1/Glucagon Dual Agonist SP-1373 Shows Superior Body and Liver Weight Loss in DIO Rodent Models Relevant to Diabetes and NASH* (Spitfire Pharma, Inc., 77th American Diabetes Association Scientific Sessions, 2017).

[22] Hansen HH (2017). Mouse models of nonalcoholic steatohepatitis in preclinical drug development. Drug Discov. Today.

[23] Laboratory, J. Body weight information for C57BL/6J (000664), https://www.jax.org/jax-mice-and-services/strain-data-sheet-pages/body-weight-chart-000664 (2020).

[24] Boland ML (2019). Towards a standard diet-induced and biopsy-confirmed mouse model of non-alcoholic steatohepatitis: Impact of dietary fat source. World J. Gastroenterol..

[25] Cariou B (2013). Dual peroxisome proliferator-activated receptor α/δ agonist GFT505 improves hepatic and peripheral insulin sensitivity in abdominally obese subjects. Diabetes Care.

[26] Cariou B, Zaïr Y, Staels B, Bruckert E (2011). Effects of the new dual PPARα/δ agonist GFT505 on lipid and glucose homeostasis in abdominally obese patients with combined dyslipidemia or impaired glucose metabolism. Diabetes Care.

[27] Flint A (2021). Randomised clinical trial: Semaglutide versus placebo reduced liver steatosis but not liver stiffness in subjects with non-alcoholic fatty liver disease assessed by magnetic resonance imaging. Aliment Pharmacol. Ther..

[28] Staels B (2013). Hepatoprotective effects of the dual peroxisome proliferator-activated receptor alpha/delta agonist, GFT505, in rodent models of nonalcoholic fatty liver disease/nonalcoholic steatohepatitis. Hepatology.

[29] Ratziu V (2016). Elafibranor, an agonist of the peroxisome proliferator-activated receptor-α and -δ, induces resolution of nonalcoholic Steatohepatitis without fibrosis worsening. Gastroenterology.

[30] Newsome P (2019). Effect of semaglutide on liver enzymes and markers of inflammation in subjects with type 2 diabetes and/or obesity. Aliment Pharmacol. Ther..

[31] Promrat K (2010). Randomized controlled trial testing the effects of weight loss on nonalcoholic steatohepatitis. Hepatology.

[32] Glass LM (2015). Total body weight loss of≥ 10% is associated with improved hepatic fibrosis in patients with nonalcoholic steatohepatitis. Dig. Dis. Sci..

[33] Marchesini G, Petta S, Dalle Grave R (2016). Diet, weight loss, and liver health in nonalcoholic fatty liver disease: Pathophysiology, evidence, and practice. Hepatology.

[34] Pyke C (2014). GLP-1 receptor localization in monkey and human tissue: Novel distribution revealed with extensively validated monoclonal antibody. Endocrinology.

[35] Knudsen LB, Lau J (2019). The discovery and development of liraglutide and semaglutide. Front. Endocrinol. (Lausanne).

[36] Nestor, J. J., Jr. In *Peptide-Based Drug Design ACS Professional Reference Book* (eds. Taylor, M. D. & Amidon, G. L.) Ch. 19, 449–471 (American Chemical Society, 1995).

[37] Nestor JJ (2009). The medicinal chemistry of peptides. Curr. Med. Chem..

[38] Lau J (2015). Discovery of the once-weekly glucagon-like peptide-1 (GLP-1) analogue semaglutide. J. Med. Chem..

[39] Larsen MT, Kuhlmann M, Hvam ML, Howard KA (2016). Albumin-based drug delivery: Harnessing nature to cure disease. Mol. Cell Ther..

[40] Millar J (1998). Shortcomings in the trough to peak ratio as a guide to the dose interval for antihypertensive drugs. J. Hum. Hypertens..

[41] Aroda VR (2018). A review of GLP-1 receptor agonists: Evolution and advancement, through the lens of randomised controlled trials. Diabetes Obes. Metab..

[42] Henderson SJ (2016). Robust anti-obesity and metabolic effects of a dual GLP-1/glucagon receptor peptide agonist in rodents and non-human primates. Diabetes Obes. Metab..

[43] Ribier, D., Tolborg, J. L., Hamprecht, D. W. & Thomas, L. Acylated glucagon analogue. United States patent US 10,336,802 B2 (2019).

[44] Kristiansen MN (2016). Obese diet-induced mouse models of nonalcoholic steatohepatitis-tracking disease by liver biopsy. World J. Hepatol..

[45] Tolbol KS (2018). Metabolic and hepatic effects of liraglutide, obeticholic acid and elafibranor in diet-induced obese mouse models of biopsy-confirmed nonalcoholic steatohepatitis. World J. Gastroenterol..

[46] Clapper JR (2013). Diet-induced mouse model of fatty liver disease and nonalcoholic steatohepatitis reflecting clinical disease progression and methods of assessment. Am. J. Physiol. Gastrointest. Liver Physiol..

[47] Kleiner DE (2005). Design and validation of a histological scoring system for nonalcoholic fatty liver disease. Hepatology.

