# Supplementary material for: Effects of ALT-801, a GLP-1 and glucagon receptor dual agonist, in a translational mouse model of non-alcoholic steatohepatitis
Source: Sci Rep. 2022 Apr 23;12:6666. doi: 10.1038/s41598-022-10577-2 (PMC9035150; doi:10.1038/s41598-022-10577-2)
Supplement: Supplementary file 1 — Supplementary Figures. [file 41598_2022_10577_MOESM1_ESM.docx]

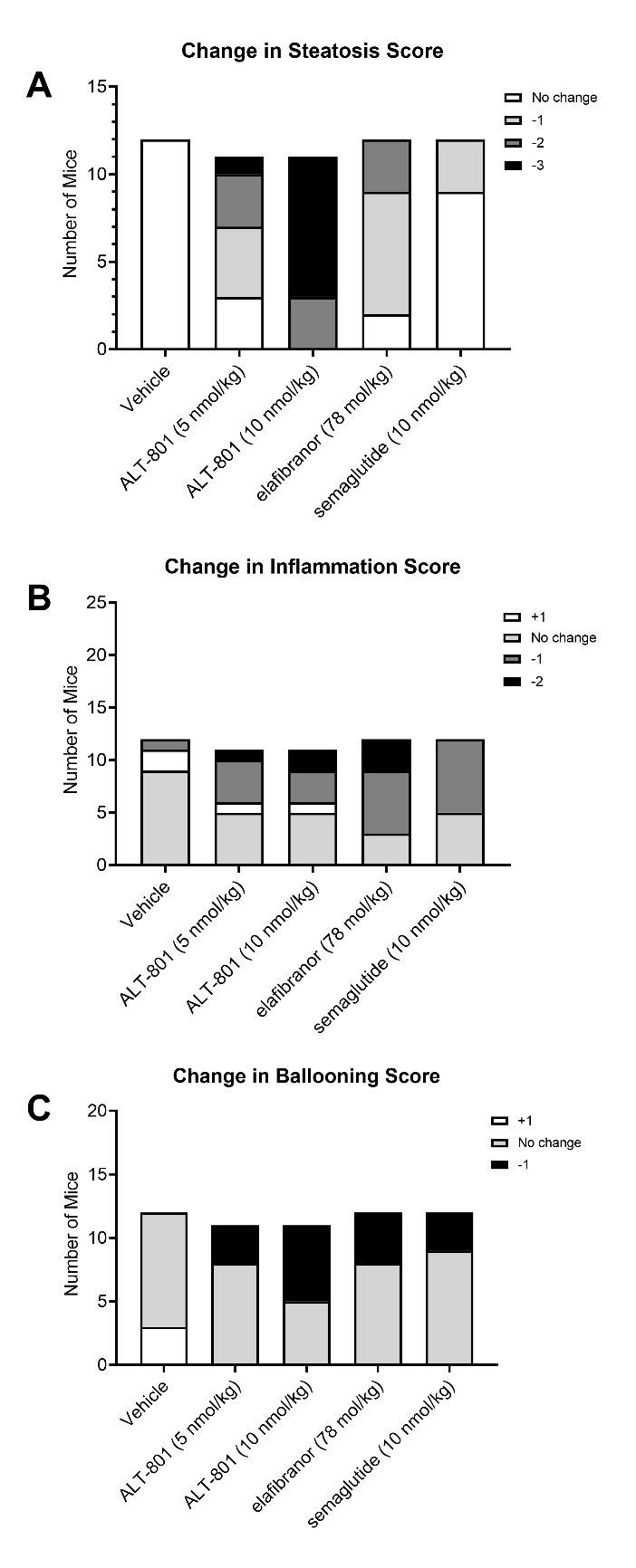


**Supplementary Fig. S1: Summary of histopathological scoring of pre- and post-study biopsies.** Change in scoring for (A) steatosis, (B) inflammation, and (C) ballooning in DIO-NASH mice.


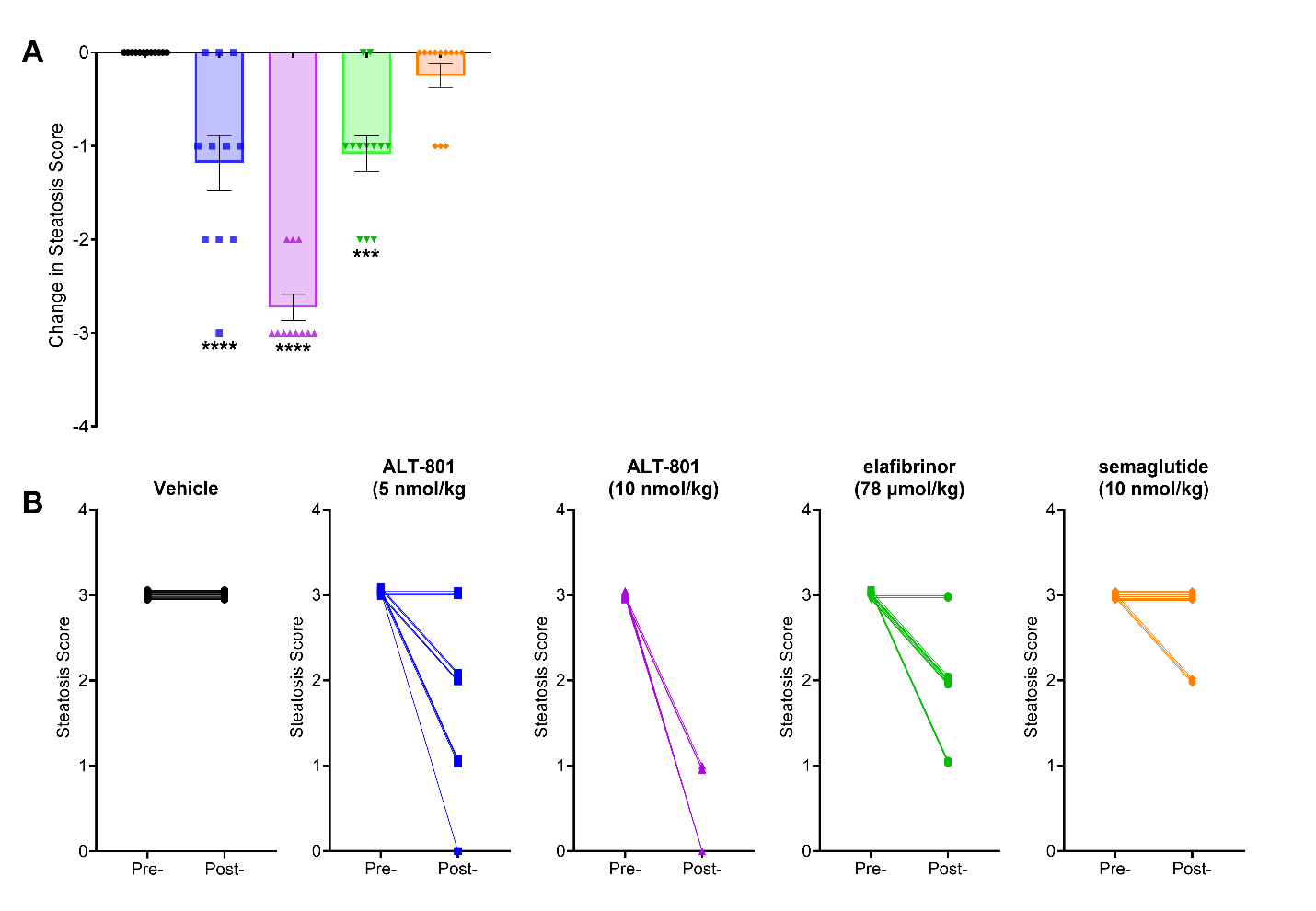


**Supplementary Fig. S2: Overview of the result of steatosis scores.** For each animal, the change from pre-study to post-study biopsy is indicated by a line. The points at each scoring step are slightly shifted to allow visual separation of the animals. This presentation is only for visualization purposes and does not reflect any difference in score. ****p<*0.001, *****p<*0.0001 vs. vehicle control.

**
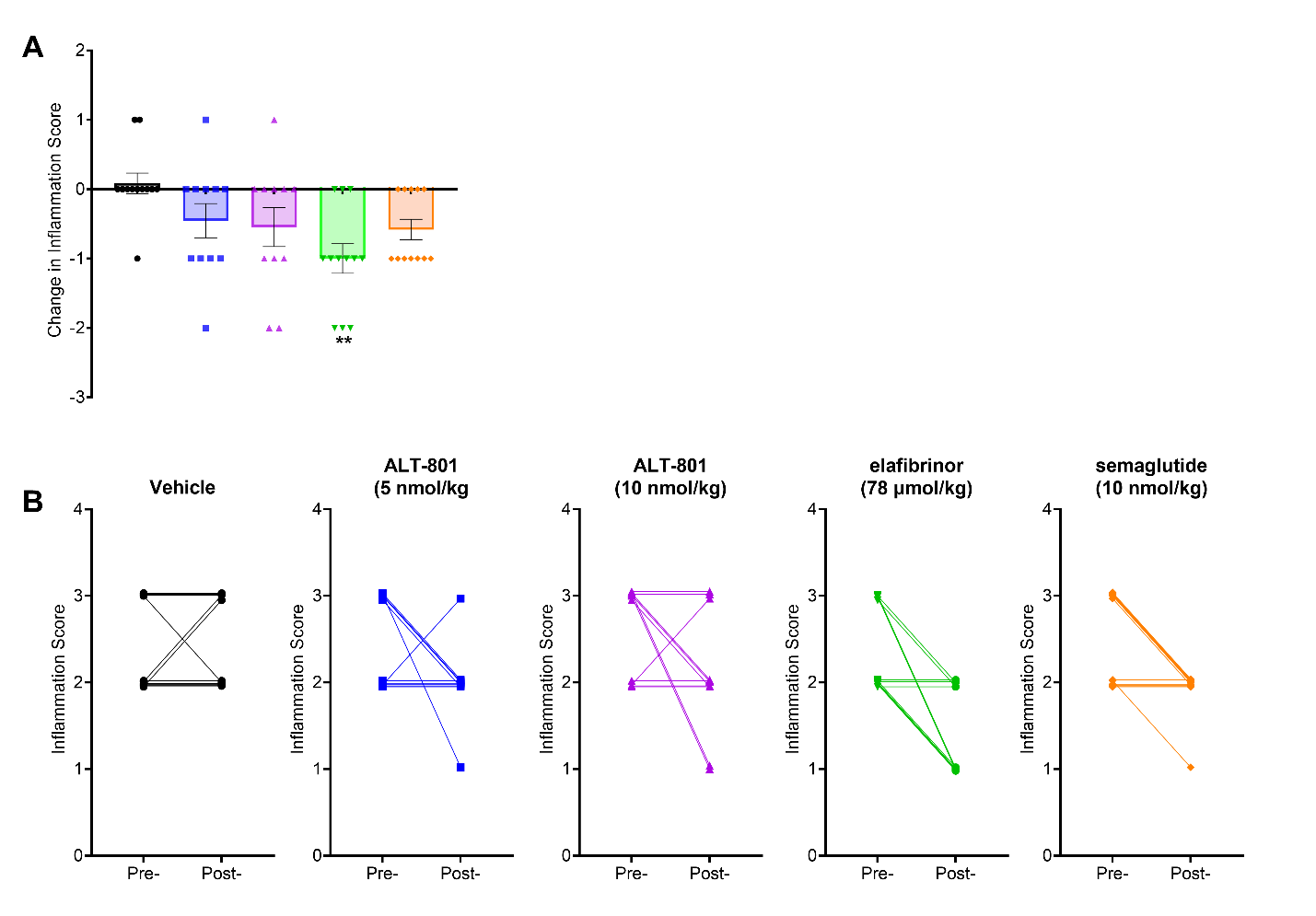
**

**Supplementary Fig. S3: Overview of the result of inflammation scores.** For each animal, the change from pre-study to post-study biopsy is indicated by a line. The points at each scoring step are slightly shifted to allow visual separation of the animals. This presentation is only for visualization purposes and does not reflect any difference in score. ***p<*0.01 vs. vehicle control.

**
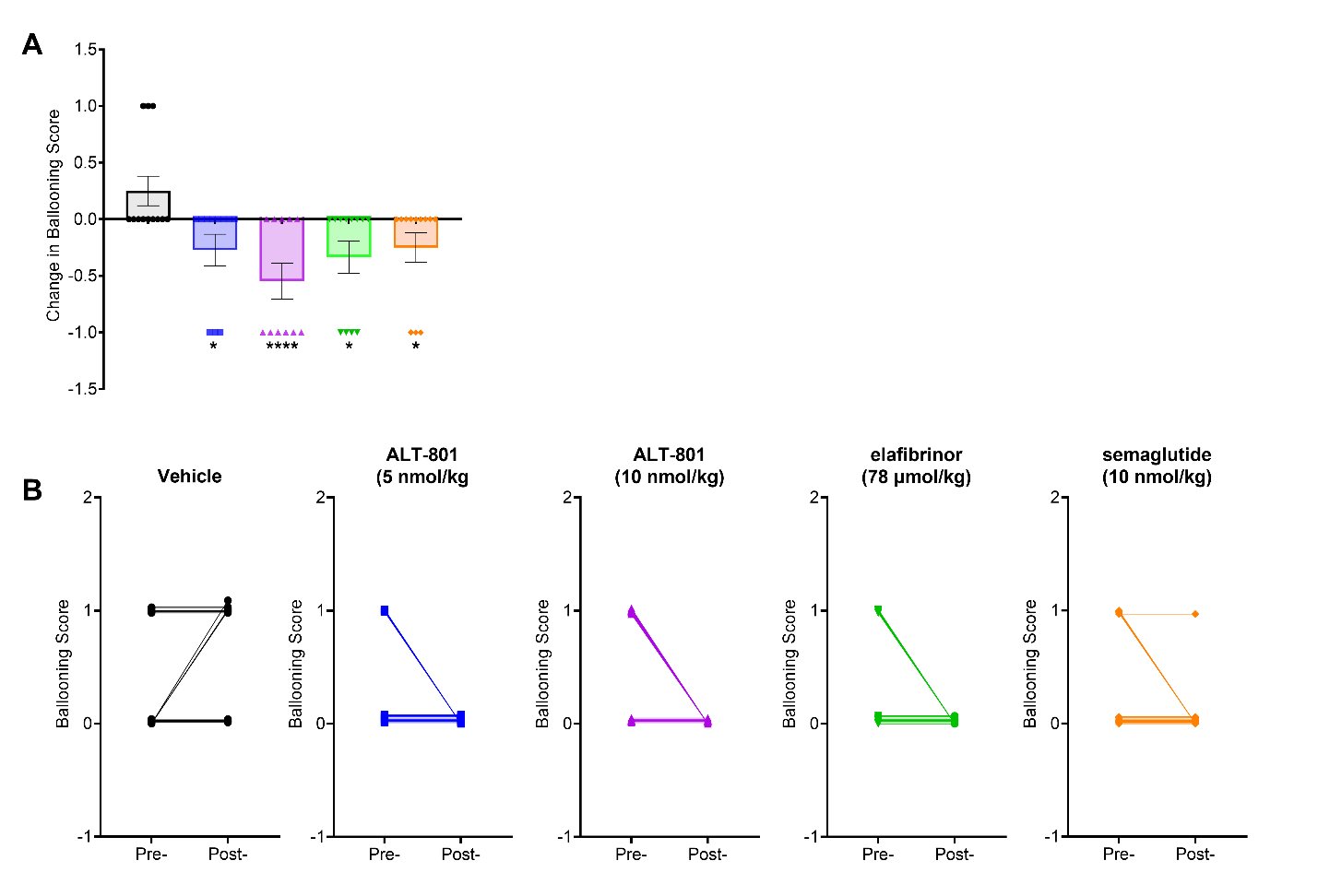
**

**Supplementary Fig. S4: Overview of the result of ballooning degeneration scores.** For each animal, the change from pre-study to post-study biopsy is indicated by a line. The points at each scoring step are slightly shifted to allow visual separation of the animals. This presentation is only for visualization purposes and does not reflect any difference in score. **p<*0.05, *****p<*0.0001 vs. vehicle control.
